# Tandem repeats discovery service (TReaDS) applied to finding novel cis-acting factors in repeat expansion diseases

**DOI:** 10.1186/1471-2105-13-S4-S3

**Published:** 2012-03-28

**Authors:** Marco Pellegrini, Maria Elena Renda, Alessio Vecchio

**Affiliations:** 1 Istituto di Informatica e Telematica, Consiglio Nazionale delle Ricerche, Pisa I-56124, Italy; 2 Dipartimento di Ingegneria dell'Informazione, Università di Pisa, Pisa I-56122, Italy

## Abstract

**Background:**

Tandem repeats are multiple duplications of substrings in the DNA that occur contiguously, or at a short distance, and may involve some mutations (such as substitutions, insertions, and deletions). Tandem repeats have been extensively studied also for their association with the class of repeat expansion diseases (mostly affecting the nervous system). Comparative studies on the output of different tools for finding tandem repeats highlighted significant differences among the sets of detected tandem repeats, while many authors pointed up how critical it is the right choice of parameters.

**Results:**

In this paper we present *TReaDS - Tandem Repeats Discovery Service*, a *tandem repeat meta search engine*. *TReaDS *forwards user requests to several state of the art tools for finding tandem repeats and merges their outcome into a single report, providing a global, synthetic, and comparative view of the results. In particular, *TReaDS *allows the user to (*i*) simultaneously run different algorithms on the same data set, (*ii*) choose for each algorithm a different setting of parameters, and (*iii*) obtain a report that can be downloaded for further, off-line, investigations. We used *TReaDS *to investigate sequences associated with repeat expansion diseases.

**Conclusions:**

By using the tool *TReaDS *we discover that, for 27 repeat expansion diseases out of a currently known set of 29, *long fuzzy tandem repeats *are covering the expansion loci. Tests with control sets confirm the specificity of this association. This finding suggests that long fuzzy tandem repeats can be a new class of cis-acting elements involved in the mechanisms leading to the expansion instability.

We strongly believe that biologists can be interested in a tool that, not only gives them the possibility of using multiple search algorithm at the same time, with the same effort exerted in using just one of the systems, but also simplifies the burden of comparing and merging the results, thus expanding our capabilities in detecting important phenomena related to tandem repeats.

## Background

### Overview on repeat expansion diseases

At present 29 diseases are classified as *repeat expansion diseases *(RE) [1-3], and the number is growing. These are mostly neurodegenerative and neuromuscolar disorders, including Huntington disease (HD), Kennedy disease (SBMA), and several types of Spinocerebral Ataxias (SCA). Since up to recently all known cases involved repeating a motif of 3 nucleotides, this class was denoted also as *trinucleotide repeat *(TNR) *expansion disease*. However, cases of repeating units with 4, 5 and 12 nucleotides have been discovered thus we talk more generally of repeat expansion diseases. Recent surveys devoted to DNA repeats [4] have extended discussion of repeat expansion disorders, while specific surveys for repeat expansion diseases can be found in [5-7].

The locus of expansion can be located in various regions of the resident gene: in the coding sequences, in the 5'- untranslated region (5'-UTR), in the 3'- untranslated region (3'-UTR), in introns and in promoter regions. Two main questions are related to the study of these diseases from a genetic point of view: (a) which mechanisms or conditions lead to the repeat expansion? and (b) how do repeat expansions result in diseases?

Only a small fraction of all the tandem repeats found in the human genome expand and result in a disease. Thus researchers have tried to identify which unusual structural features favor such expansion, and found a propensity to forming hairpins (or other structures, such as: quadruplex-like structures, H-DNA and sticky DNA) as a key mechanism leading to expansion. Several studies also tried to identify cis-regulating elements that do favor the onset of the above structural features and of the expansion. Our study falls in this category and proposes *long fuzzy tandem repeats *as a novel cis-regulating element for repeat expansion, thus contributing to investigating question (a).

### Cis-acting factors for TNR instability

Several papers tackle the problem of determining cis-acting factors associated with loci of TNR instability. In particular one quite studied factor is the proximity and orientation of DNA replication initiation regions (IR) w.r.t. the TNR instability locus [8,9]. In [8] the position of the DNA replication initiation region for three TNR diseases loci (HD, SCA7, and SBMA) is analyzed, and a correlation pattern is proposed. The role of flanking regions to the expansion locus (EL) has been analyzed in literature. For example, close proximity of the TNR locus to CpG-rich regions has been noticed in some cases (10 diseases) [10]. The presence of the transcription factor binding site (TFBS) CTCF has been discovered in the flanking region for SCA7 [11]. An association between HD and an haplogroup (with SNPs not necessarily in the flanking sequences) is described in [12]. Note that such studies identify cis-acting factors relevant only for a few RE diseases.

Fuzzy tandem repeats (FTRs) have been recently proposed as a new genomic feature worth of study [13,14]. Informally, FTRs are tandem repeats with high divergence (30-40%) between the repeating units and the consensus motif. At the best of our knowledge, up to now the hypothesis that Fuzzy TRs can act as cis-elements for human diseases was not explored in the literature. Interestingly we have found FTRs in almost all the RE disease independently from the specific repeating motif, coding/non-coding characterization, etc. Thus FTRs may be seen as a "generic" cis-acting factor that may in particular cases interact with other cis-acting factors specific for the single protein/disease.

Analysis of TNR instability has been conducted also in other model species, e.g. *Saccaromyces cerevisiae *[15] and *Escherichia Coli *[16].

### Role of hairpins

In several cases it has been noticed that the TNR RNA coding sequences tend to form hairpin structures [17-19] or RNA-DNA hybrids such as R-loops [20]. This is relevant in particular for the TNR located in the transcribed sections of DNA. These results on hairpin are obtained via experiments *in vitro*, usually involving a relatively short repeating sequence (a trinucleotide unit repeated 16 or 17 times) and a promoter sequence. In these experiments the role of the native flanking regions is factored out or in some cases different (non-native) flanking sequences are used. Evidence of hairpin formation with the natural flanking sequence for SCA3, SCA6 and Dentatorubropallidoluysian atrophy (DRPLA) is reported in [21]. Notice thus that, although hairpin formation is an important mechanism to explain trinucleotide instability, one cannot infer the presence of a FTR just from the tendency to form hairpin (or other) RNA structure in vitro. The relationship of FTR and hairpin formation is at the moment unclear and it is an open area for future research, as in this stage we are interested in establishing FTR as a potential cis-regulatory element, rather than exploring the precise mechanisms of the action.

### PolyQ repeats

For the subfamily of nine polyQ repeat diseases the corresponding polyglutammine peptide has been studied in some detail [22,23]. Such studies are important for determining the toxicity mechanism of the mutant proteins, however they explain the onset of the disease only after the expansion at the DNA locus occurs. In particular the pathogenic length of the polyQ chain is a specific trait of each disease. A list of such diseases is reported in table 1.

**Table 1:** Table of polyglutammine diseases.

Disease code	Disease name	Gene code	Normal repeats	Pathogenic repeats
DRPLA	Dentatorubropallidoluysian atrophy	ATN1	6 - 35	49 - 88
HD	Huntington's disease	HTT (Huntingtin)	10 - 35	35+
SBMA	Kennedy disease (Spinobulbar muscular atrophy)	HS-AR	9 - 36	38 - 62
SCA1	Spinocerebellar ataxia Type 1	ATXN1	6 - 35	49 - 88
SCA2	Spinocerebellar ataxia Type 2	ATXN2	14 - 32	33 - 77
SCA6	Spinocerebellar ataxia Type 6	CACNA1A	4 - 18	21 - 30
SCA7	Spinocerebellar ataxia Type 7	ATXN7	7 - 17	38 - 120
SCA17	Spinocerebellar ataxia Type 17	TBP	25 - 42	47 - 63
SCA3	Machado-Joseph disease (Spinocerebellar ataxia Type 3)	ATXN3	12 - 40	55 - 86

Table of polyglutammine diseases. The table reports: disease code and full name, associated gene, ranges of healthy and pathogenic repeat numbers.

### PolyA repeats

A second class of repeat expansion diseases involve repetitions of the imperfect GCN triplets that encode the Alanine amino acid. Such REs are characterized by relative low copy numbers (both in the normal and expanded states). In addition the expanded polyA repeats are stable both in the somatic and intergenerational transmission, unlike polyQ repeat expansions. A list of such diseases is reported in table 2.

**Table 2:** Table of polyalanine diseases.

Disease code	Disease name	Gene code	Normal repeats	Pathogenic repeats
BPES	Blepharophimosis-ptosis-epicanthus inversus syndactyly	FOXL2	14	19-24
HPE5	Holoprosencephaly 5	ZIC2	15	25
CCHS	Congenital failure of autonomic control	PHOX2B	20	25-33
ISSX	X-linked infantile spasm syndrome	ARX	16	27
MRGH	X-linked mental retardation with isolated growth hormone deficiency	SOX3	15	22-26
CCD	Cleidocranial dysplasia	RUNX2	17	27
HFGS	Hand-foot-genital syndrome	HOXA13	18	24-26
SPD1	Synpolydactyly 1	HOXD13	15	22-29
OPMD	Oculopharyngeal muscular dystrophy	PABPN1	10	11-17

Table of polyalanine diseases. The table reports: disease code and full name, associated gene, ranges of healthy and pathogenic repeat numbers.

### Non-polyQ and non-polyA repeats

Non-polyQ and non-polyA expanding repeats may have motifs of length 3,4,5, and 12. They may be located in several sections of the gene sequence. A list of such diseases is reported in table 3.

**Table 3:** Table of non-polyglutammine, non-polyalanine diseases.

Disease code	Disease name	Gene	Motif	Location	Normal repeats	Pathogenic repeats
FRAXA	Fragile X syndrome	FMR1	CGG	5'-UTR	6 - 53	230+
FXTAS	Fragile Xassociated tremor/ataxia syndrome	FMR1	CGG	5'-UTR	6 - 53	55-200
FRAXE	Fragile XE mental retardation	AFF2	GCC	5'-UTR	6 - 35	200+
FRDA	Friedreich's ataxia	FXN	GAA	Intr.	7 - 34	100+
DM1	Myotonic dystrophy type	DMPK	CTG	3'-UTR	5 - 37	50+
DM2	Myotonic dystrophy type 2	ZNF9	CCTG	Intr.	27-	75+
SCA10	Spinocerebellar ataxia Type 10	ATXN10	ATTCT	Intr.	10-29	280+
SCA12	Spinocerebellar ataxia Type 12	PPP2R2B	CAG	5'-UTR	7 - 28	66 - 78
EPM1	Progressive myoclonus epilipsy	CSTB	(*C*)_4_*G*(*C*)_4_*GCG*	Prom.	2-3	60+
HDL-2	Huntington diesease-like	JPH3	CAG/CTG	3'-UTR	66-	66+
SCA8	Spinocerebellar ataxia Type 8	ATXN8OS	CTG	3'-UTR	16 - 37	110 - 250

Table of non-polyglutammine and non-polyalanine diseases. The table reports: disease code and full name, associated gene, repeating unit genic region, ranges of healthy and pathogenic repeat numbers.

### Fuzzy tandem repeats as potential cis-regulatory elements in repeat expansion disorders

In [14] we noticed that the locus associated with the unstable trinucleotide repeat in the Frataxin protein mRNA coding sequence (whose abnormal expansion is cause of Frederich's ataxia) was included in a much longer fuzzy TR, detected using the proposed TRStalker system.

The present research originated from the hypothesis that this fact (a long fuzzy TR covering the unstable locus) could be observed in a large number of trinucleotide repeat disorders. Consequently, FTR could be exposed as a novel cis-regulatory element not yet studied in literature.

We employ the tool *TReaDS *in order to quickly collect and organize the output of several TR finding algorithms into a single easy to read report in support to this hypothesis.

### Tools for finding tandem repeats

Tandem repeats (TRs) of different forms (satellites, microsatellites, minisatellites) have been studied extensively because of their role in several biological processes. In fact, TRs are privileged targets in activities such as fingerprinting or tracing the evolution of populations [24,25]; several diseases, disorders and addictive behaviors are linked to specific TRs loci [26]; the role of TRs has been also studied within coding regions [27] and in relation to gene functions [28].

The scope and depth of the research on TRs have been boosted by the availability of efficient non-trivial algorithms for finding TRs, even when mutations occur with non-negligible probability. Tandem Repeat Finder (TRF) [29], CRISPRFinder [30], mreps [31], Reputer [32], Approximate Tandem Repeat Hunter (ATRHunter) [33], TandemSWAN [13], and Tread [34] are some examples of currently operational systems that can be accessed via a web interface.

Comparative studies [13,35], for the case of short TRs with high percentages of substitutions, report significant differences among the sets of TRs that can be detected by using different tools. Moreover, in [35] it is highlighted how critical it is the choice of parameters. Thus, biologists could highly benefit from a tool that gives them the possibility of simultaneously querying multiple systems and getting a global, comparative and synthetic view of the results, with the same effort one would exert in using just one of the systems.

In this paper we present *TReaDS - Tandem Repeats Discovery Service*, a *TRs meta search engine *that forwards the user requests to different tandem repeat finding services and aggregates the results. More in detail, *TReaDS *allows the user to (*i*) simultaneously run different algorithms on the same data set, (*ii*) choose manually, for each algorithm, a different parameter settings, or express her/his request in a simple and concise way (exact or approximate, short or long TRs), delegating to *TReaDS *the burden of choosing the right choice of parameters for all the systems, and (*iii*) get back a report that can be downloaded for further, off-line, investigations.

*TReaDS *is currently interfaced with five services based on different algorithmic principles and techniques, thus a joint use of them is likely to lead to increased precision. In order to improve the quality of service *TReaDS *offers to its users, we plan to add to *TReaDS *other existing systems and new ones at the time they become available.

## Methods

*TReaDS *is a web application, and it has been completely developed by using Java-based technologies. In particular, a pool of Servlets takes care of handling the users' request (file upload, parameter settings, search), and collects the results generated by the systems involved in the query. *TReaDS *merges the results received from the external services and produces the final report with the support of the JasperReports publicly available libraries [36] On the client side there is no special requirement: just a standard browser and a viewer (suitable for the report format selected by the user).

*TReaDS *has the proper structure of a meta search engine, with options for changing the set of parameters of each algorithm, and for choosing the output format. The publicly available web tools for finding tandem repeats currently supported by *TReaDS *are: ATRHunter [37] mreps [38] TandemSWAN [39] and TRF [40]. *TReaDS *is interfaced with the version of these tools available on-line. Note that a binary version of these systems can be also downloaded and, in some cases, there are some small differences between the web-based and the downloadable versions, especially in terms of the number of parameters that can be customized. Furthermore *TReaDS *supports TRStalker [14], an algorithm developed by our team aimed at finding long fuzzy TRs under weighted edit distance.

### TReaDS input/output

**Figure 1:**
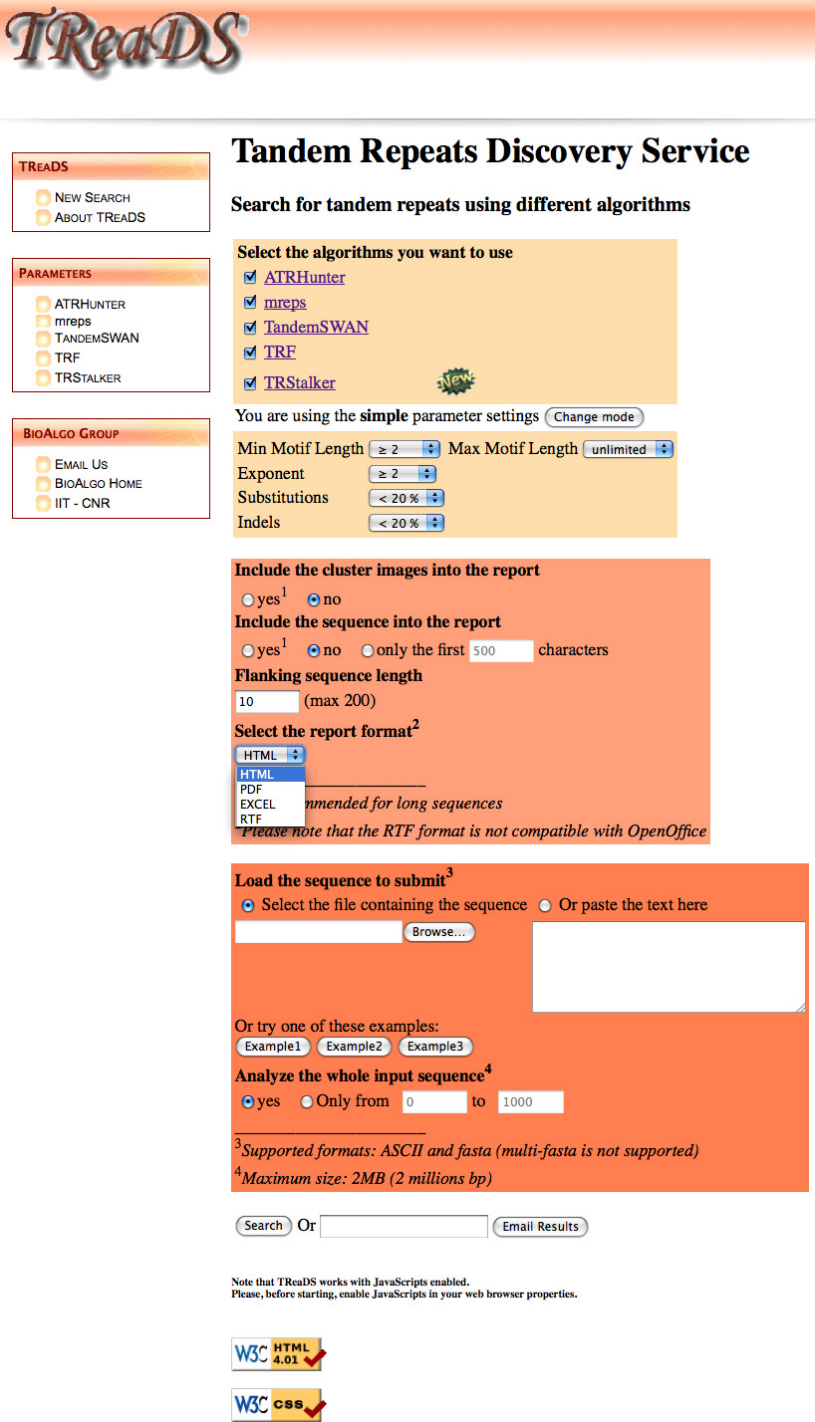
**TReaDS: main page of the graphics user interface**. The main page of the graphics user interface allows setting the input parameters. This page has sub-sections for: algorithms selection, parameters of the tandem repeats to be reported, style of the output report, and input sequence.

The main page of *TReaDS *is essentially composed of four sections: (1) *Algorithms*, (2) *Parameter Settings*, (3) *Report*, and (4) *Sequence *(see Figure 1).

In the **Algorithms **section it is possible to choose any combination of the supported systems.

In the **Parameter setting **section *TReaDS *provides two ways to set the parameters for the chosen systems: (*i*) the *simple mode*, where it is possible to specify the kind of TRs to look for, by setting the minimum and maximum motif length, the minimum exponent (i.e. the number of repetitions), and the maximum percentages of allowed substitutions and in/dels (insertions and deletions); (*ii*) the *advanced mode*, where the user can run each system with manually selected parameters, if she wants a fine-grained control over the settings.

In the **Report **section the user:

1. decides if she wants in the final report a graphical visualization of the found TRs;

2. chooses if the input sequence (or a part of it) must be included into the final report;

3. sets the length of the *flanking sequence*; and

4. chooses the final report format among the available ones: HTML, Excel, PDF, and RTF.

In the **Sequence **section it is possible to submit a sequence as a file, or to paste it in a given text area; furthermore the user can chose if the whole sequence or just a part of it must be analyzed. *TReaDS *takes as input either a FASTA or plain text genomic sequence. The size limit for an input sequence corresponds to the present limit of ATRHunter: 2Mbp.

The user can decide to wait on-line for the result or to receive them via email by providing a valid email address.

**Figure 2:**
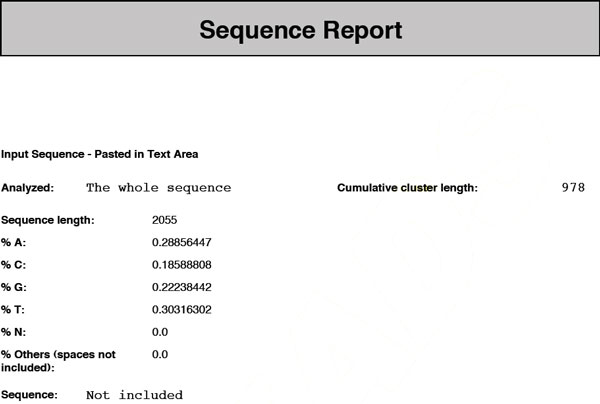
**TReaDS: example of the sequence sub-report**. The sequence sub-report of the output report gives basic statistics on the nucleotide frequencies of the input sequence and the total length of the clusters of tandem repeats reported.

**Figure 3:**
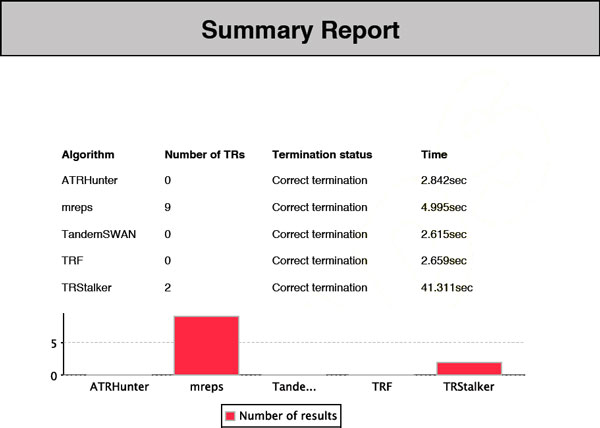
**TReaDS: example of the summary sub-report**. The summary sub-report of the output report gives the termination codes for each algorithm and basic statistics on the number of tandem repeats detected by each algorithm.

**Figure 4:**
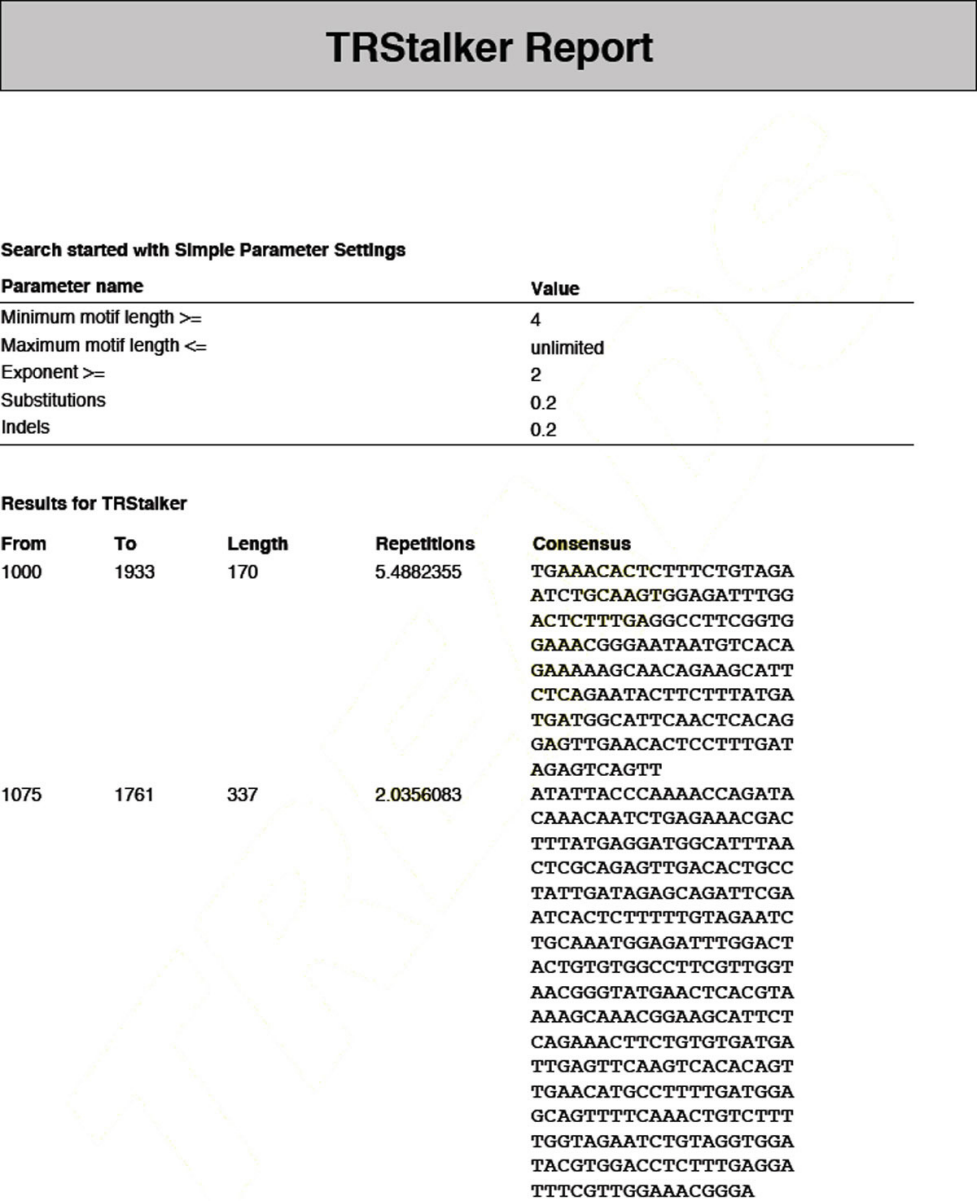
**TReaDS: example of an algorithm sub-report**. The algorithm sub-report of the output report lists separately the tandem repeats found by each algorithm and their basic features.

**Figure 5:**
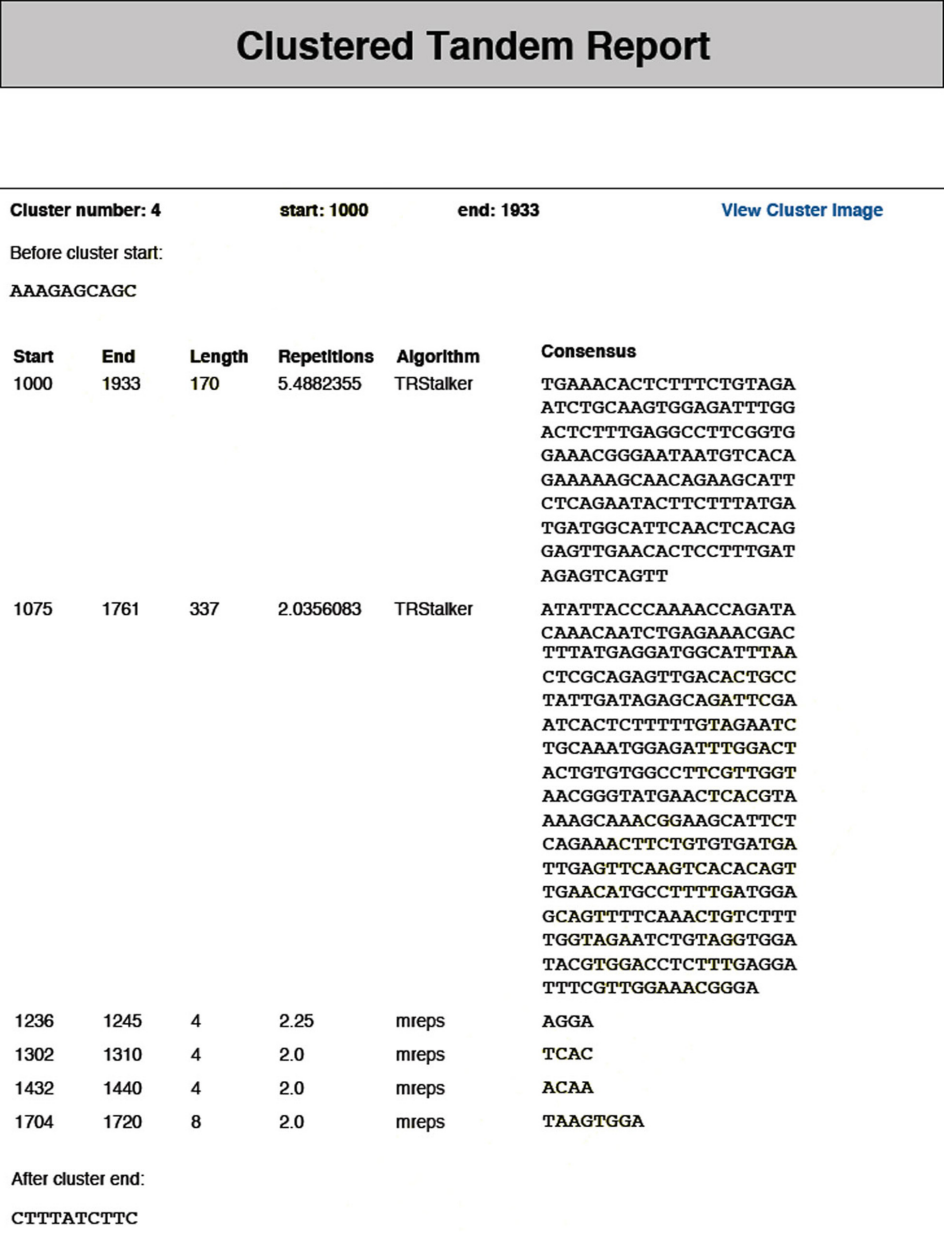
**Example of a cluster returned by TReaDS**. The clusters sub-report of the output report lists all tandem repeats organized in clusters of overlapping tandem repeats. For each cluster its beginning and end positions are reported, and the constituent tandem repeats.

**Figure 6:**
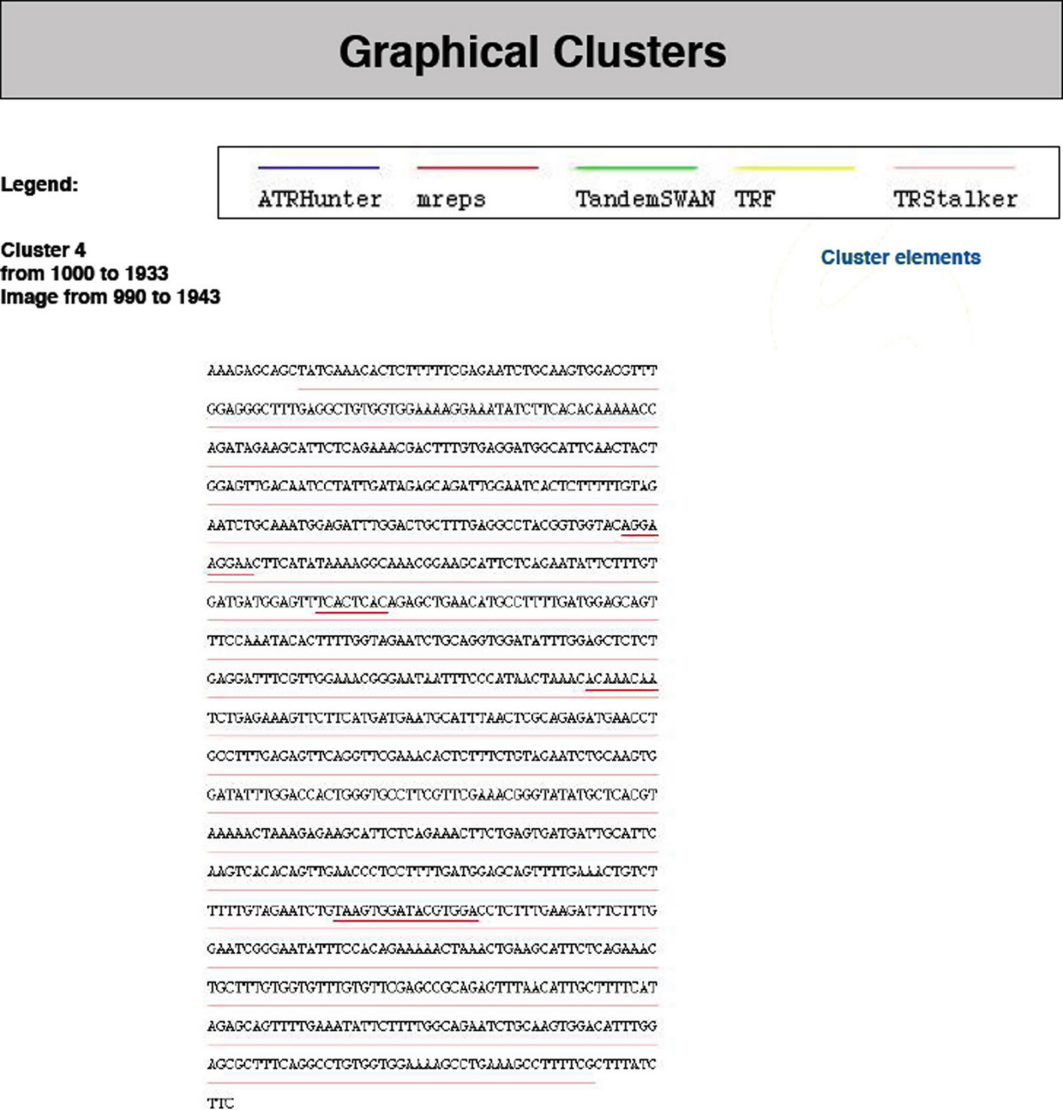
**A cluster returned in graphical format**. Example of a cluster returned by *TReaDS*: the cluster is showed in a graphical format, with the original sequence opportunely underlined in the position of the found TR, and with different colors, each corresponding to the particular queried algorithm returning the TR.

Once the responses coming from the TR finding services have been received, *TReaDS *merges the results and produces a report containing the following sub-reports:

• **Sequence sub-report**. The sequence sub-report contains the sequence, if requested, and some information such as length and distribution of the different bases (see Figure 2).

• **Summary sub-report**. The summary sub-report contains, for each system involved in the query, the algorithm name, the number of TRs found, whether the connection has been successful (if not, the type of error encountered is reported), and the response time. It is also provided a chart that shows a comparison of the systems (the comparison is simply based on the number of TRs found) (see Figure 3).

• **Algorithm sub-reports**. There is one algorithm sub-report for each system included in the search process (see, for instance, Figure 2). It contains the detail of the parameters used and the list of the TRs found by the specific algorithm, including their initial position, length, number of repetitions, and consensus. In case of *advanced mode *search the parameters are those the user set for the given algorithm, while in case of *simple mode *search the global parameters given as input are reported (see Figure 4).

• **Clusters sub-report**. *TReaDS *merges the results of all algorithms to give a global view of them by identifying overlapping TRs. Two TRs overlap if they share one or more positions in the sequence. The overlapping relation is an equivalence relation thus it allows us to partition the found TRs into groups that we call *clusters*. Such clusters are reported in the *clusters sub-report *(see Figure 5). Graphically, a cluster covers a contiguous segment of the input sequence without gaps. The report contains a list of all *clusters *found. For each cluster the following information is included: flanking sequence (if requested), starting and ending positions of the covered segment, list of TRs that form the cluster, and some details for each TR (starting and ending position, length, number of repetitions, consensus). If the user has chosen to include the images in the final report, it is also possible to view each cluster in a graphical form (see Figure 6).

## Results

### Experimental methodology

The relevant sequences have been downloaded from PubMed (See NCBI codes in Additional file 1) and the position of the expansion locus identified via reference to the relevant literature for the target disease. For sequences up to 10000 nt the whole sequence has been analyzed. For longer sequences a sub-sequence in the range -5000 +5000 nt centered on the expansion locus has been analyzed. The tool *TReaDS *has been set with 5 algorithms; the parameter setting is reported in table 4.

**Table 4:** Parameters for TReaDS used in the experiments.

Parameter name	Value
Minimum motif length	10
Maximum motif length	unlimited
Minimum repeat number	2
Maximum substitution	20%
Maximum indel	20%

Parameters for *TReaDS *used in the experiments..

First, we run *TReaDS *and by inspecting the output returned it is possible to identify the longest TR covering the expansion locus. In a second phase, for each analyzed sequence, the algorithm that found a covering FTR has been tuned so to possibly find a better fuzzy TR (with a longer motif, and lower error level), while minimizing the measure of the union of fuzzy TRs of the same type in that sequence.

In most cases a single covering FTR has been found. In one case (SCA10) two partially overlapping FTRs cover the expansion locus. The FTRs found have copy number roughly between 2 and 3 in most cases. In principle, a FTR containing an EL may arise from a large self-overlapping of the EL segment in the FTR. Thus we need to show that such self-overlapping does not influence our data. Simple consideration based on the ratio of the lengths of the FTR and EL segments imply that no self-overlapping can occur when the ratio is greater or equal to 2. For a ratio 1.8 at most the overlap can be of the order of 10% of the length of the EL.

We also measure the total length of the regions of the sequence covered by FTR of the same type (same motif length or longer, and same percentage of error) as the one identified as covering the expansion locus. The ratio of this length and the length of the sequence gives a conservative estimate to the probability that a randomly chosen position in the sequence is covered by a FTR of the type considered. The value of such probability is quite small for almost all of the sequences, resulting in an average probability over all the sequences associated to repeat expansion diseases of 0.12.

### Experiments with repeat expansion sequences

The list of the major diseases due to repeat expansion are taken from [2,3].

An important subfamily is composed of polyglutammine diseases (polyQ) since the repeated triplet motif is the codon CAG, in a coding region, that encodes the glutamine (Q) amino acid (see table 1 and 5). A second subfamily is the family of polyanaline (polyA) expansion disease, where the expanding motif is formed by triplets GCN (see table 2 and 6). Other diseases are classified as non-polyQ and non-polyA and are listed in tables 3 and 7.

**Table 5:** Table of fuzzy tandem repeats for PolQ TR.

Gene code	Seq length	Cover	TR-beg	TR-end	FTR-beg	FTR-end	FTR/TR	Cover/Length
ATN1	4367	206	1687	1743	1646	1751	1.875	0.047
HTT	13481	196	197	265	196	367	2.514	0.014
HS-AR	4314	377	1286	1354	1224	1391	2.455	0.087
ATXN1	10636	4237	1560	1646	1500	1718	2.534	0.398
ATXN2	4712	401	658	726	629	748	1.75	0.085
CACNA1A	8641	579	7186	7224	7160	7425	6.973	0.067
ATXN7	7242	443	641	670	576	743	5.758	0.061
TBP	1921	305	451	564	389	636	2.185	0.158
ATXN3	10000(*)	-	943	984	-	-	-	-

Table of fuzzy tandem repeats for PolQ TR. The table reports: gene code, sequence length, length of the region covered by FTRs, TR expansion begin and TR expansion end, FTR begin and FTR end, ratio of FTR length over TR length, ratio of region covered by FTRs over total sequence length. (*) indicates that a subsequence has been analyzed.

**Table 6:** Table of covering fuzzy tandem repeats for polyalanine TR.

Gene code	Seq length	Cover	TR-beg	TR-end	FTR-beg	FTR-end	FTR/TR	Cover/Length
FOXL2	9900	5000	6079	6115	6082	6258	4.888	0.505
ZIC2	11701	677	8385	8429	8304	8534	5.227	0.057
PHOX2B	11889	187	7940	7993	7830	8015	3.490	0.015
ARX	19255	1039	7252	7299	7199	7424	4.787	0.053
SOX3	9074	1114	5700	5744	5584	6191	13.795	0.122
RUNX2	10000 (*)	766	99435	99485	99310	99488	3.560	0.076
HOXA13	10227	658	5375	5428	5328	5493	3.113	0.064
HOXD13	10135	279	5256	5300	5025	5304	6.340	0.027
PABPN1	12976	1337	6286	6303	6278	6405	7.470	0.103

Table of covering fuzzy tandem repeats for polyalanine TR. The table reports: gene code, sequence length, length of the region covered by FTRs, TR expansion begin and TR expansion end, FTR begin and FTR end, ratio of FTR length over TR length, ratio of region covered by FTRs over total sequence length. (*) indicates that a subsequence has been analyzed.

**Table 7:** Table of covering fuzzy tandem repeats for non-polyglutammine and non-polyalanine TR.

Gene code	Seq length	Cover	TR-beg	TR-end	FTR-beg	FTR-end	FTR/TR	Cover/Length
FMR1	46137	3415	5061	5171	4983	5168	1.681	0.074
AFF2	16800	595	5021	5062	4958	5429	11.487	0.035
FXN	2465	723	2185	2212	2036	2414	14.000	0.293
DMPK	2465	273	2304	2363	2213	2365	2.576	0.110
ZNF9	23153	5462	16312	16387	16264	17088	10.986	0.235
ATXN10(**)	50000(*)	1301	128559	128628	28543	28654	1.608	0.026
PPP2R2B	5120	703	2088	2366	1842	2363	1.874	0.137
CSTB	9429	3098	4899	4935	4472	5019	15.194	0.328
JPH3	10000(*)	807	35581	35746	35476	35755	1.690	0.080
ATXN8OS	39541	-	37142	37216	-	-	-	-

Table of covering fuzzy tandem repeats for non-polyglutammine and non-polyalanine TR. The table reports: gene code, sequence length, length of the region covered by FTRs, TR expansion begin and TR expansion end, FTR begin and FTR end, ratio of FTR length over TR length, ratio of region covered by FTRs over total sequence length. (*) indicates that a subsequence has been analyzed. (**) indicates two overlapping FTRs.

### Specificity of fuzzy tandem repeats for genes with CAG-encoded polyglutammine

In order to test the specificity of the association of covering fuzzy TRs with repeat expansion loci we have analyzed a sample of genes with long CAG-encoded polyglutammine (more than 6 repeating units). We have chosen this subclass since it has been extensively studied in literature. The statistics for this type of repeats have been collected in [6] that lists 148 sequences in ORF regions (out of a total of 718), and [41] listing 64 polyQ genes. We have examined the first 25 entries of the list in [6] having CAG repeats in ORF regions. Entries no more present in NCBI Nucleotide databases have been replaced by the newer version of the same gene when possible; entries for the same gene have been merged. Thus we have examined a total of 17 sequences in tables 8 and 9.

**Table 8:** Table of covering fuzzy tandem repeats for a sample of CAG-encoded polyglutammine that have been investigated for possible connections to pathologies.

Gene code	Tri-repeat position	FTR position	References
RAI1	1300+39	1290-1368	[41,42]
DACH1	846+42	830-926	[43,44]
(TNRC3) MAML3	2220+36,	2187-2292,	[41,45]
	2667+24,	2628-2698,	
	3030+24	2960-3053	
NRG2	302+18, 329+24	227-401	[46,52]

Table of covering fuzzy tandem repeats for a sample of CAG-encoded polyglutammine that have been investigated for possible connection to pathologies. The table reports: gene code, location of the polyQ locus, location of the fuzzy TR if existing, relevant references.

**Table 9:** Table of covering fuzzy tandem repeats for a sample of CAG-encoded polyglutammine.

Gene code	Tri-repeat position	FTR position	References
NFAT5	1497+18	-	[41]
vascular endothelial cadherin 2	4739+18	-	
PRDM8	1865+18	-	
PRDM10	3327+27	-	[41]
ATBF1-A	10262+21	-	
USP7	208+21	-	
IRS1	2049+18	-	
(ATBF1) ZFHX3	10262+21	-	
FBX11	90+21	-	
PCQAP	611+18, 711+18, 831+36	607-652, 712-869	[41]
(DRIL2) ARID3B	214+24	-	
POU3F2	594+18	516-618	[41]
PALM2-AKAP2	1738+18	-	

Table of covering fuzzy tandem repeats for a sample of CAG-encoded polyglutammine. The table reports: gene code, location of the polyQ locus, location of the fuzzy TR if existing, relevant references.

Four sequences have been investigated in literature for their potential role in diseases (table 8).

Polymorphism of the the CAG repeat in protein RAI1 has been found to influence the onset age in patient affected by the spinocerebellar ataxia type 2 (SCA2) [42]. Data shown in [43,44] indicate a genetic linkage of the chromosomal region containing the gene DACH with many developmental disorders affecting limbs, kidneys, eyes, and ears, although specific causality and mechanisms still need to be elucidated. The gene MAML3 is shortlisted in [45] for further study in disease associations, based on comparing the conservation patterns among human, mouse and rat genomes. The human neuregulin-2 (NRG2) gene has been evaluated for a possible association with the Charcot-Marie-Tooth disease [46]. Since the pathogenic status of these repeats is still unclear we exclude them from further analysis.

For the remaining 13 sequences (table 9) we have found evidence of a covering Fuzzy TR in 2 cases (15%).

### Specificity of fuzzy tandem repeats for genes with pathological SNPs

In this section we explore the issue of the specificity of FTR covering mutation loci linked to pathological conditions. We explore two different types of mutations, the first one is due to single nucleotide substitutions (SNP). The data base dbSNP (Human Build 135) [47,48] lists as of today, 1835 records of pathogenic SNPs for Homo Sapiens sequences. We have selected a sample (See Additional file 1) of such sequences and analyzed them using *TReaDS*. Results reported in table 10 show that out of 43 pathogenic SNPs in 14 sequences, only 2 are covered by a long FTR (14%).

**Table 10:** Table of pathogenic SNPs in Homo sapiens from dbSNP and covering fuzzy tandem repeats.

Gene/Protein	Seq length	Num. path. SNP	Covered by FTR	FTR
FZD6	3806	2	0	-
NSDHL	1581	2	0	-
GJB1	1623	10	0	-
IDS	1437	5	0	-
IDS	5832	2	0	-
SLC16A2	4396	2	0	-
NSDHL	1581	2	0	-
ABCB7	2404	2	0	-
TIMM8A	1459	2	0	-
UBA1	3544	3	0	-
FLNA	8533	2	2	[1000- 3946]
MED12	6985	1	0	-
PRPS1	2156	4	0	-
ARSE	2220	4	0	-

Table of pathogenic SNPs in Homo sapiens from dbSNP and covering fuzzy tandem repeats. The table reports: gene/protein code, sequence length, number of pathogenic SNP, number of pathogenic SNPs covered by FTR, FTR [Begin - End] if existing.

### Specificity of fuzzy tandem repeats for genes with pathological in/dels

The data base dbSNP (Human Build 135) lists, as of today, 391 records of pathogenic short in/dels for sequences of Homo Sapiens. We have selected a sample of such sequences (See Additional file 1) and analyzed them using *TReaDS*. Data in table 11 show that for 67 pathogenic in/dels only 9 are covered by FTR (13%).

**Table 11:** Table of pathogenic in/dels in Homo sapiens from dbSNP and covering fuzzy tandem repeats.

Gene/Protein	Seq length	Num. path. in/dels	Covered by FTR	FTR	
CFTR/MRP	1000 (*)	2	0	-	
OTC	1000 (*)	3	2	[117 - 883],	[429 - 569]
OTC	1647	30	0	-	
HS mitochondrion	16569	1	0	-	
NSDHL	1581	2	0	-	
GJB1	1623	2	1	[319-373]	
SLC16A2	4396	2	0	-	
SLC6A8	3580	2	0	-	
CACNA1F	6080	1	0	-	
FLNA	8533	1	1	[280 329]	
KCNQ2	3158	21	5	[162-275]	[1666-1691]
				[2188-2214]	[2654-2728]

Table of pathogenic in/dels in Homo sapiens from dbSNP and covering fuzzy tandem repeats. The table reports: gene/protein code, NCBI code for the analyzed sequence, sequence length, codes of pathogenic in/del, number of pathogenic in/dels covered by FTR, FTR [Begin - End] if existing. (*) indicates that the analysis has been done on a subsequence of length 1000 centered on the position of each in/del.

## Conclusions

### Results on repeat expansion diseases

We have found that for the current set of 29 repeat expansion diseases in 27 cases (93%) there is a long fuzzy TR covering the expansion locus. The ratio of the length of the fuzzy TR to the expansion locus ranges from a minimum of 1.608 and a maximum of 15.194. Also the specificity of the association has been investigated for the set of genes with CAG-encoded polyglutammine tracts, for pathogenic SNPs, for pathogenic in/dels, and for the non-pathogenic sections of the sequences. This specificity analysis shows that in just about 15% of the control cases there is an association to fuzzy TRs. These preliminary results indicate that fuzzy TRs may be an important novel cis-element that influences the instability of the expansion locus. However, a more in depth analysis and consideration of causal mechanisms involved is needed to confirm the correlation between fuzzy TRs and RE diseases.

### The power of TReaDS

As large scale studies are being pursued, it is important to facilitate the use of the TR search engines publicly available. In the literature, the comparison of several TR finding tools highlighted significant differences among the sets of results. Other work made evident the importance of tuning the parameters of operation. In this paper we presented *TReaDS*, a web application which provides a single user interface and enables a simultaneous application of different techniques on the same data set. With *TReaDS *the user can express the characteristics of her request through a simple and unified interface, or she can customize the set of parameters of each system. The user gets back a report that contains a global and comparative view of the results. The report can be downloaded for a deeper off-line investigation. This way, *TReaDS *allows to harness the power of different web-based TR search engines with a minimal effort.

Furthermore, merging and comparing the outcome of different search tools on the same data can be useful for gaining higher confidence that all the relevant TRs in the data set have been found.

To the best of our knowledge *TReaDS *is the first meta search engine for tandem repeats and there is no similar and comparable system freely available.

### Future work

The database *TRbase *[49] maintains an annotated correspondence between genes known to be involved in some disease and the tandem repeats in their DNA sequence (detected with TRF [29]). For the class of repeat expansion diseases a direct causal link between TRs and the onset of the disease is known. As future work we plan to analyze the correlation between other diseases (or disease classes) and the presence and type of fuzzy TRs, using *TReaDS*, in order to suggest hypothesis on possible roles for fuzzy TRs in that context.

In this paper we studied those trinucleotide expansion (and repeat expansion) leading to the manifestation of diseases. However, polymorphic microsatellites and ministatellites are very common in the human genome (as well as in all eukaryote genomes), thus one could advance the hypothesis that FTR may have a facilitating role in such polymorphisms (independently from the manifestation of a pathology). Testing this far-reaching hypothesis which is our next objective, is far from trivial since comprehensive maps of polymorphic/monomorphics TRs for the human genome, (even restricted the coding regions) are just being produced [50,51].

## List of abbreviations

DRPLA: Dentatorubropallidoluysian atrophy; EL: Expansion locus; FTR: Fuzzy tandem repeats; HD: Huntington disease; IR: Initiation region; polyA: polyalanine; polyQ: polyglutammine; RE: Repeat expansion; SCA: Spinocerebral ataxia; TFBS: Transcription factor binding site; TNR: Trinucleotide repeat; TR: Tandem repeat; TReaDS: Tandem repeats discovery service.

## Competing interests

The authors declare that they have no competing interests.

## Availability and requirements

• **Project name: ***TReaDS*

• **Project home page: **http://bioalgo.iit.cnr.it/treads

• **Operating system(s): **Platform independent

• **Programming language: **Java

• **Other requirements: **JavaScripts Enabled (on the client side)

• **License: **Lesser General Public License (LGPL)

• **Any restrictions to use by non-academics: **None, *TReaDS *is a web application free and open to all users

## Authors' contributions

AV conceived of the application tool, participated in its design and development, and helped to draft the manuscript. MER participated in the design and development of the application, performed the testing and debugging phases, performed experiments, and helped to draft the manuscript. MP conceived the application of *TReaDS *to repeat expansion sequences, performed experiments, drafted the final manuscript and exercised general supervision. All authors read and approved the final manuscript.

## Supplementary Material

Additional file 1 **"Tandem repeats discovery service (*TReaDS*) applied to finding novel cis-acting factors in repeat expansion diseases - supplementary information --" contains NCBI codes of analyzed sequences and dbSNP codes for the analyzed SNPs and in/dels**. Click here for file
