# Supplementary material for: Tandem repeats discovery service (TReaDS) applied to finding novel cis-acting factors in repeat expansion diseases
Source: BMC Bioinformatics. 2012 Mar 28;13(Suppl 4):S3. doi: 10.1186/1471-2105-13-S4-S3 (PMC3303744; doi:10.1186/1471-2105-13-S4-S3)
Supplement: Additional file 1 — "Tandem repeats discovery service (TReaDS) applied to finding novel cis-acting factors in repeat expansion diseases - supplementary information --" contains NCBI codes of analyzed sequences and dbSNP codes for the analyzed SNPs and in/dels. [file 1471-2105-13-S4-S3-S1.pdf]

# Tandem repeats discovery service (TReaDS) applied to finding novel cis-acting factors in repeat expansion diseases – supplementary information —

Marco Pellegrini[1], M. Elena Renda*[1] , Alessio Vecchio[2]

[1]Istituto di Informatica e Telematica, Consiglio Nazionale delle Ricerche, Pisa I-56124 , Italy
[2]Dipartimento di Ingegneria dell'Informazione, Università di Pisa, Pisa I-56122 , Italy

Email: Marco Pellegrini - marco.pellegrini@iit.cnr.it; M. Elena Renda*- elena.renda@iit.cnr.it; Alessio Vecchio - a.vecchio@iet.unipi.it;

*Corresponding author

| Disease code | Gene | NCBI code |
|---|---|---|
| DRPLA | ATN1 | NM_001007026.1 |
| HD | HTT | NM_002111.6 |
| SBMA | AR | NM_000044.2 |
| SCA1 | ATXN1 | NM_000332.3 |
| SCA2 | ATXN2 | NM_002973.3 |
| SCA3 | ATXN3 | NM_004993.5 |
| SCA6 | CACNA1A | NM_000068.3 |
| SCA7 | ATXN7 | NM_000333.3 |
| SCA17 | TBP | NM_003194.4 |

Table 1: Table of sequence NCBI codes for PolyQ TR.

| Disease code | Gene | NCBI code |
|---|---|---|
| BPES | FOXL2 | NG_012454.1 |
| HPE5 | ZIC2 | NG_007085.2 |
| CCHS | PHOX2B | NG_008243.1 |
| ISSX | ARX | NG_008281.1 |
| MRGH | SOX3 | NG_009387.1 |
| CCD | RUNX2 | NG_008020.1 |
| HFGS | HOXA13 | NG_008181.1 |
| SPD1 | HOXD13 | NG_008137.1 |
| OPMD | PABPN1 | NG_008239.1 |

Table 2: Table of sequence NCBI codes for PolyA TR.

| Disease code | Gene | NCBI code |
|---|---|---|
| FRAXA/FXTAS | FMR1 | NG_007529.1 |
| FRAXE | AFF2 | NG_016313.1 |
| FRDA | FXN | U43748.1 |
| DM1 | DMPK | NM_001081560.1 |
| DM2 | ZNF9 | NG_011902.1 |
| SCA8 | ATXN8OS | NR_002717.2 |
| SCA10 | ATXN10 | NG_016212.1 |
| SCA12 | PPP2R2B | AF152102.1 |
| EPM1 | CSTB | NG_011545.1 |
| HDL-2 | JPH3 | AF429315.1 |

Table 3: Table of sequence NCBI codes for non PolyA non PolyQ TR.

| Gene | NCBI code |
|---|---|
| NFAT5 | AF163836 |
| vascular endothelial cadherin 2 | AF240635 |
| PRDM8 | NM_020226 |
| PRDM10 | NM_020228 |
| (TNRC3) MAML3 | NM_018717.4 |
| ATBF1-A | L32832 |
| USP7 | NM_003470 |
| IRS1 | NM_005544 |
| DACH1 | NM_004392 |
| (ATBF1) ZFHX3 | NM_006885 |
| NRG2 | NM_004883 |
| FBX11 | AF176706 |
| PCQAP | NM_015889 |
| (DRIL2) ARID3B | NM_006465 |
| RAI1 | NM_030665.3 |
| POU3F2 | NM_005604 |
| PALM2-AKAP2 | NM_007203 |

Table 4: Table of Table of sequence NCBI codes for a sample of sequences containing CAG-encoded polyglutammine.

| Gene/Protein | NCBI code | SNP names |
|---|---|---|
| FZD6 | NM_003506.3 | rs151339003, rs151339002 |
| NSDHL | NM_015922.2 | rs137853863, rs116840822 |
| GJB1 | NM_001097642.2 | rs116840819, rs116840818, rs116840817, rs116840816, rs116840815, rs104894901, rs104894825, rs104894824, rs104894822, rs104894812 |
| IDS | NM_006123.4 | rs113993955, rs113993954, rs113993953, rs113993949, rs113993945 |
| IDS | NM_001166550.1 | rs113993951, rs113993946 |
| SLC16A2 | NM_006517.3 | rs104894939, rs104894938 |
| NSDHL | NM_015922.2 | rs104894909, rs104894901 |
| ABCB7 | NM_004299.3 | rs80356714, rs80356713 |
| TIMM8A | NM_004085.3 | rs80356560, rs80356559 |
| UBA1 | NM_153280.2 | rs80356547, rs80356546, rs80356545 |
| FLNA | NM_001456.3 | rs80338841, rs80338837 |
| MED12 | NM_005120.2 | rs80338759 |
| PRPS1 | NM_002764.3 | rs80338732, rs80338731, rs80338676, rs80338675 |
| ARSE | NM_000047.2 | rs80338714, rs80338713, rs80338711, rs80338710 |

Table 5: Table of pathogenic SNPs in Homo sapiens from dbSNP. The table reports: gene/protein code, NCBI code for the analyzed sequence, codes of pathogenic SNP.

| Gene/Protein | NCBI code | In/Del names |
|---|---|---|
| CFTR/MRP | NM_001171.4 | rs111113625, rs72664225 |
| OTC | - | rs111060773, rs72558496, rs111060774 |
| OTC | NM_000531.5 | rs72558496 rs72558469, rs72558460, rs72558459, rs72558458, rs72558457, rs72558453, rs72558452, rs72558439, rs72558438, rs72558434, rs72558422, rs72556285, rs72556264, rs72556255, rs72556251, rs72554357, rs72554355, rs72554354, rs72554353, rs72554343, rs72554334, rs72554314, rs72554313, rs72552299, rs72552298, rs72552297, rs67870245, rs67839039, rs67294956 |
| HS mitochondrion | NC_012920.1 | rs111033319 |
| NSDHL | NM_015922.2 | rs121909834, rs121909833 |
| GJB1 | NM_001097642.2 | rs116840823, rs116840820 |
| SLC16A2 | NM_006517.3 | rs113994166, rs113994164 |
| SLC6A8 | NM_005629.3 | rs80338740, rs80338739 |
| CACNA1F | NM_005183.2 | rs80359870 |
| FLNA | NM_001456.3 | rs80338840 |
| KCNQ2 | NM_172108.3 | rs118192246, rs118192245, rs118192244, rs118192243, rs118192242, rs118192241, rs118192231, rs118192230, rs118192227, rs118192222, rs118192213, rs118192212, rs118192210, rs118192198, rs118192197, rs118192196, rs118192192, rs118192191, rs118192189, rs118192188, rs118192187 |

Table 6: Table of pathogenic in/dels in Homo sapiens from dbSNP and covering fuzzy tandem repeats. The table reports: gene/protein code, NCBI code for the analyzed sequence, codes of pathogenic in/del.
